# A blocking antibody against anti-Müllerian hormone restores ovulation and normal androgen levels in a spontaneous rat model of polycystic ovary syndrome

**DOI:** 10.1016/j.ebiom.2025.105716

**Published:** 2025-04-18

**Authors:** Chrystèle Racine, François Fraissinet, Stefania Tolu, Tony Pereira, Stéphanie Gil, Anne Badel, Danielle Bailbé, Bruno Fève, Jamileh Movassat, Richard Cate, Nathalie di Clemente

**Affiliations:** a Sorbonne Université, Inserm, Centre de Recherche Saint-Antoine (CRSA), UMR_S938, Paris 75012, France; b Institut Hospitalo-Universitaire ICAN, Paris 75013, France; c Université Paris Cité, Paris 75013, France; d Department of General Biochemistry, Rouen University Hospital, Rouen 76000, France; e Université Paris Cité, BFA, UMR8251, CNRS, Paris 75013, France; f Department of Endocrinology, Hôpital Saint-Antoine, APHP, Paris 75012, France; g Department of Chemistry, Boston University, Boston, MA, USA

**Keywords:** Anti-Müllerian hormone, Blocking antibody, Polycystic ovary syndrome, Goto-Kakizaki rats, Ovulation, Androgens

## Abstract

**Background:**

Polycystic Ovary Syndrome (PCOS), the leading cause of infertility worldwide, is characterised by oligo-anovulation, hyperandrogenism, polycystic ovarian morphology and high Anti-Müllerian hormone (AMH) levels, associated with severe metabolic disturbances. However, the role of AMH in the physiopathology of this syndrome remains poorly understood and strategies to block its effects have never been investigated in animal models of PCOS.

**Methods:**

We used Western-blotting, ELISA and gene reporter approaches to evaluate the blocking efficacy, interspecificity and mechanism of action of an antibody against human AMH, Mab22A2. Then, we investigated the ability of a rat version of Mab22A2, rMab22A2, to alleviate reproductive dysfunction in Goto-Kakizaki (GK) rats, which spontaneously exhibit all the features of women with PCOS.

**Findings:**

We showed that Mab22A2 was interspecific, did not prevent AMH from binding to its receptor and was able to block the effects of AMH in gonadal cell lines. In addition, treatment of anovulatory GK rats with rMab22A2 reduced their bioavailable serum AMH levels and normalised their androgen concentrations. Finally, this treatment also induced ovulation in 84% of the rats and resulted in 66% of pregnancies.

**Interpretation:**

Our results show that AMH is a major driver of reproductive and hormonal dysfunction in PCOS and provide proof of concept that a blocking antibody against AMH can reverse the major reproductive dysfunction observed in PCOS, opening up promising avenues for the treatment of patients with PCOS.

**Funding:**

10.13039/501100001677Inserm, 10.13039/501100019125Sorbonne University, 10.13039/501100015760Inserm Transfert, the French Endocrine Society and the 10.13039/501100009187Medical Research Foundation (grant agreement n°EQU201903007868).

**Research in context**

**Evidence before this study**

The inhibitory role of AMH on oestradiol production and on terminal folliculogenesis has long been documented in a physiological context. However, although AMH levels are elevated in women with PCOS and correlated with the severity of the syndrome, there has been only indirect evidence that AMH is involved in the hyperandrogenism and ovulation defects observed in these patients. Furthermore, no strategy has been investigated to neutralise the effects of high AMH levels in vivo in a PCOS-like model.

**Added value of this study**

We have identified and characterised a monoclonal antibody against human AMH, Mab22A2, capable of blocking its signalling pathway and steroidogenic target genes in gonadal cell lines. After producing Mab22A2 in a rat format, we tested different immunotherapy protocols in anovulatory GK rats, a spontaneous rodent model of PCOS that displays all the reproductive and metabolic features of this condition. We were able to find a treatment protocol that reduced androgen levels and restored ovulation and pregnancy in these rats.

**Implications of all the available evidence**

This study has shown that the high levels of AMH in GK rats are responsible for their hyperandrogenism and ovulation defects. In addition, this work provides proof of concept that an anti-AMH blocking antibody could reverse the reproductive dysfunction observed in women with PCOS, and an innovative therapy for these patients.

## Introduction

Polycystic ovary syndrome (PCOS) is the leading cause of infertility in women, affecting 8–13% of women worldwide.^1^ It is diagnosed by the presence of at least two of the three Rotterdam criteria: oligo-anovulation, clinical or biochemical hyperandrogenism and polycystic ovarian morphology on ultrasound. As serum anti-Müllerian hormone (AMH) concentrations are elevated in patients with PCOS and are related to the number of antral follicles detected by ultrasound, this parameter has also recently been proposed as an alternative to ultrasound. Women with PCOS have other reproductive disorders such as high serum levels of Luteinising Hormone (LH) and an increased risk of developing gestational diabetes, pre-eclampsia and preterm birth. In addition, these patients have varying degrees of metabolic abnormalities, such as obesity, which ranges from 20 to 80% depending on the ethnicity, insulin resistance and dyslipidaemia, leading to an increased risk of developing type 2 diabetes (T2DM) and cardiovascular disease.^2^ Importantly, most of these features are passed on to the children of women with PCOS, with 60–70% of their daughters developing the syndrome.^3^

However, the aetiology and pathophysiology of this very complex syndrome are still not well understood. A growing body of evidence suggests that a combination of genetic, epigenetic and environmental factors, particularly the maternal environment, contribute to the reproductive and metabolic abnormalities observed in women with PCOS.^2^^,^^3^ Among the factors involved in the pathophysiology of PCOS, androgens, insulin and AMH, have been recognised as playing a central role. Because many women with PCOS are obese, the first line of treatment to manage the altered fertility of these patients is lifestyle intervention, with diet and exercise, followed by pharmacological therapy to supplement women with Follicle-Stimulating Hormone (FSH), to allow the increased number of follicles to mature and induce ovulation.

AMH is a member of the transforming growth factor-beta family expressed in women by granulosa cells of growing follicles, from the onset of folliculogenesis until menopause.^4^ AMH is translated as a homodimeric precursor, containing an N-terminal pro-domain and a smaller C-terminal mature domain. The AMH precursor undergoes an obligatory cleavage at monobasic sites between the two domains, but the N- and C-terminal homodimers remain associated in a non-covalent complex (AMH procomplex). The AMH procomplex binds to the AMH-specific type II receptor AMHR2, leading to the dissociation of the N-terminal pro-domain, the interaction of AMHR2 with type I serine/threonine kinase receptors, and the phosphorylation of the transcription factors Smad1, 5, or 8. In recent years, AMH has emerged as an important regulator of folliculogenesis capable of acting at different levels of the hypothalamic-pituitary-gonadal axis.^5^ Indeed in rodents, AMH has been shown to inhibit primordial follicle assembly^6^ and recruitment,^7^ as well as the responsiveness of growing follicles to FSH,^8^ more specifically the FSH-dependent induction of aromatase, the enzyme responsible for converting androgens into oestrogens, and to prevent follicular atresia.^9^ In addition, AMH can act directly on the hypothalamus to stimulate GnRH neurons, thereby inducing LH secretion by the pituitary gland.^10^

Cumulative evidence suggests that AMH may be involved in the ovulation defects of women with PCOS. Indeed, anovulatory women with PCOS have AMH levels that are 18-fold higher than normo-ovulatory women with PCOS,^11^ with AMH levels correlating with the severity of the condition, particularly with respect to anovulation.^12^ In addition, the dysregulation of AMH and AMHR2 expression by LH has been observed in granulosa cells from anovulatory women with PCOS, but not in granulosa cells from normo-ovulatory patients with PCOS.^13^ It is thought that in women with PCOS, elevated AMH levels have a more pronounced inhibitory effect on follicular sensitivity to FSH, leading to a failure of dominant follicle selection, and hence follicular arrest.^14^ In addition, high levels of AMH may contribute to increased levels of androgen in women with PCOS, and to the androgenisation of their foetuses during pregnancy, leading to the development of PCOS in adulthood, and consequently to transgenerational inheritance of this syndrome.^15^^,^^16^ However, treatments to specifically block the potential effects of AMH have not been tried in the context of PCOS.

One limitation to a better understanding of the pathophysiology of PCOS and the development of innovative therapeutic strategies for women with PCOS, has been the lack of a rodent model in which PCOS develops spontaneously.^17^^,^^18^ We found that the inbred Goto-Kakizaki (GK) rat strain, a well-known model of lean T2DM, also exhibited the 3 Rotterdam criteria as well as elevated AMH and LH levels.^19^ In addition, like women with PCOS, the oestrous cycles of GK rats became irregular between 3.5 and 5 months, and by 6 months, 75% of GK rats were anovulatory.

In this article, we investigated the ability of a laboratory-derived antibody directed against the C-terminal bioactive domain of human AMH to block its effects in vitro, its mechanism of action and its interspecificity. We also investigated the blocking capacity of this antibody in vivo and we assessed whether reducing bioavailable AMH levels in anovulatory GK rats, could normalise their androgen levels, and restore their oestrous cycles.

## Methods

### Reagents

AMH proteins have been described previously.^20^^,^^21^ Briefly, AMH precursor and uncleavable AMH with a mutation (R451T) at the monobasic cleavage site, were purified by immunoaffinity chromatography from culture medium of Chinese Hamster Ovary (CHO) cells transfected with the wild-type human AMH gene or with the R451T mutant AMH cDNA, respectively.^22^ AMH precursor contains approximately 95% AMH precursor and 5% cleaved AMH. Uncleavable AMH, has been assessed as 0% cleaved by SDS-PAGE. Completely cleaved AMH corresponding to the non-covalent AMH complex or AMH procomplex, was prepared by plasmin digestion of AMH and has been assessed as 100% cleaved by SDS-PAGE. C-terminal AMH was purified from the AMH procomplex.^20^ The soluble fusion protein containing the extracellular domain of human AMHR2 and the fragment crystallisable region (Fc) part of human IgG1 (AMHR2-Fc) was produced in HEK293E cells as previously described and purified from conditioned medium using Protein A Sepharose and size exclusion chromatography.^23^ The monoclonal antibodies (Mab) used in this study were previously prepared by immunisation of female BALB/c mice with human AMH precursor and validated: Mab22A2^24^; Mab10.6.^20^ Smad1-Gal4, Smad5-Gal4 and Gal-Luc plasmids were kindly provided by Dr. Azeddine Atfi (Inserm UMR_S938, Paris) and validated in.^25^ Cyp19a1 (aromatase gene) and steroidogenic acute regulatory protein (StAR) reporter genes were gifts from Drs JoAnne S Richards (Baylor College of Medicine, Houston) and Stephane Fabre (INRAE Toulouse), respectively, and validated in.^26^^,^^27^

### Western-blotting

Smad phosphorylation was determined as previously described.^28^ Briefly, mouse Sertoli SMAT-1 cells^29^ were seeded at approximately 50% density into 6-well tissue culture plates in Dulbecco’s modified Eagle’s medium (DMEM) (Life Technologies, Rockville, MD) containing 10% foetal bovine serum (FBS; Life Technologies), 100 U mL^−1^ penicillin, and 100 μg mL^−1^ streptomycin (Life Technologies). The next day, the cells were washed, medium without FBS was added for 1 h (h), and AMH was added in culture medium without serum for 30 min (min). The cells were washed and lysed in 200 μL of lysis buffer (20 mM Tris-HCl, pH 7.5, 150 mM NaCl, 1% (v/v) Triton X-100) containing a mixture of proteinase inhibitors (Sigma–Aldrich, St Louis, MO) and a phosphatase inhibitor cocktail (Calbiochem Merck Biosciences, Darmstadt, Germany). The lysates were cleared by centrifugation and supernatants were analysed by SDS-PAGE followed by western blotting with a rabbit anti-phospho (P)-Smad1, 5, 8 antibody (1:1000; 13820 (RRID: AB 2493181), Cell Signalling Technology, Ozyme, Montigny-le-Bretonneux, France) and a goat anti-rabbit IgG antibody conjugated to streptavidin-horseradish peroxydase (HRP) (1:5000; 111-035-045 (RRID: AB_2337938), Jackson ImmunoResearch Laboratories, West Grove, PA). The membranes were stripped and reprobed with a mouse anti-alpha-tubulin monoclonal antibody (1:10000; clone B-5-1-2, T5168 (RRID: AB_477579) Sigma–Aldrich). The level of P-Smad1, 5, 8 in each sample was determined by densitometric analysis (Image J) of the bands corresponding to P-Smad1, 5, 8 (56 kDa) and α-tubulin (50 kDa), and the fold increase of P-Smad1, 5, 8 compared to control medium.

### Cell culture and transfection assays

SMAT-1^29^ and KK1 (RRID: CVCL 6663,^30^) cell lines were cultured at 37 °C under 5% CO2 in DMEM or DMEM/F-12 (Life Technologies), respectively, containing 10% FBS (Life Technologies), 100 U mL^−1^ penicillin, and 100 μg mL^−1^ streptomycin (Life Technologies). Twenty-four hours before transfection, cells were seeded in 24-well plates at 7.5 × 10^4^ cells per well. Twenty-four hours later, cells were co-transfected using lipofectamine Plus reagent (Life Technologies) with 250 ng/well (w) of Gal4-Smad5 and Gal4-luc plasmids or Cyp19a1 or StAR reporter genes, and 12.5 ng/w of pRLTK vector (E2241, Promega, Madison, WI) as a control for transfection efficiency. For KK1 cells, the mouse AMHR2 cDNA (62.5 ng/w) was co-transfected with the other cDNAs. After the transfection, cells were treated with either control medium, C-terminal AMH (2 nM), AMH precursor (7.14 nM) or AMH procomplex (7.14 nM) alone, or preincubated for 1 h in the presence of a 0.2- to 100-fold mass excess of Mab22A2, or Mab10.6. Twenty-four hours later, cells were washed twice with 1× Phosphate-Buffered Saline (PBS) (Gibco, ThermoFisher Scientifc, Waltham, USA), and lysed for 20 min with rocking in 125 μL of passive lysis buffer (Promega) per well. Twenty μL were assayed for Firefly and Renilla luciferase activity according to the manufacturer’s instructions (Dual Luciferase kit, Promega) using a TriStar luminometer (EG&G Berthold, Thoiry, France). Results were expressed as the ratio of Firefly to Renilla luciferase activity. Experiments were performed at least 3 times in triplicate or quadruplicate.

### ELISAs

The conditions for ELISAs using Mab10.6 coated on plates have been previously described.^21^ The conditions for an ELISA using AMHRII-Fc have also been previously described.^23^ Briefly, Nunc Maxisorp ELISA plates (Invitrogen, Carlsbad, CA) were coated overnight at 4 °C with a goat anti-human Fc antibody (10 μg mL^−1^; 109-005-098 (RRID: AB_2337541), Jackson ImmunoResearch). After washing with water and blocking with 1% bovine serum albumin (BSA) (A-7906; Sigma–Aldrich) and 1% goat serum (Life Technologies) in PBS, AMHR2-Fc was added at a concentration of 0.75 μg mL^−1^ and incubated for 1 h. After washing with PBS, human AMH procomplex or AMH present in the serum of one bovine, one ovine, and one mouse sample was serially diluted down the plate and incubated for 2–4 h. After washing with PBS/0.05% Tween-20, biotinylated Mab22A2 (0.5 μg mL^−1^) was added and incubated for 1 h, followed by a 1 h incubation with streptavidin conjugated to HRP (1:3000; 016-030-084 (RRID: AB_2337238); Jackson ImmunoResearch). After washing, tetramethylbenzidine substrate (Pierce, ThermoFischer Scientific, Villebon-sur-Yvette, France) was added to each well, reactions were quenched by adding 2M sulphuric acid and absorbances were read at 450 nm. All the experiments were performed 3 times.

### Sequencing of the variable regions of Mab22A2

Sequencing of the variable regions of Mab22A2 was performed by the Biotem company (Apprieu, France). Briefly, total RNA was extracted from the hybridoma cells using TRIzol Reagent (Ambion, ThermoFischer Scientific) according to the manufacturer’s instructions. Reverse transcription was performed using the Superscript IV Reverse Transcriptase kit (Invitrogen Carlsbad) and OligodT to transcribe total mRNA into cDNA. PCR amplification of antibody variable regions was then performed using the NEB Q5 Hot start High fidelity enzyme (New England Biolab, Evry-Courcouronnes, France) and specific custom primer KIT for VH, VLκ and VLλ (Biotem). Five μL of each PCR reaction was run on a 1% agarose gel in Tris-Acetate-EDTA buffer at 90 V and positive PCR products were Sanger sequenced using the appropriate primer. Sequence analysis using Snapgene (Dotmatics, Boston, USA) and IMGT (Montpellier, France) tools allowed the confirmation and annotation of the sequence of the two variable domains.

### Production of Mab22A2 in the rat format

Based upon the sequences of the variable regions of Mab22A2, the Evitria company (Zürich, Suisse) performed gene design and optimisation for the expression of Mab22A2 in the rat format using in-house algorithms. The cDNA was synthesised using traditional phosphoramidite chemistry and verified by Sanger sequencing. To reduce Fc receptor interaction, N234A, L234A and P329G substitutions were introduced into the constant domain https://pubmed.ncbi.nlm.nih.gov/27578889/. Evitria cloned the corresponding cDNA into the Evitria vector system using conventional (non-PCR based) cloning techniques. The Evitria vector plasmids were genetically synthesised. Plasmid DNA was prepared under low endotoxin conditions based on anion exchange chromatography. DNA concentration was determined by measuring absorbance at a wavelength of 260 nm. The Mab22A2 variable region sequences were verified by Sanger sequencing (with up to two sequencing reactions per plasmid depending on the size of the cDNA). Evitria used suspension-adapted CHO-K1 cells (originally obtained from ATCC (RRID:CVCL_0214, Manassas, USA) and adapted to serum-free growth in suspension culture at Evitria) for production. The seed was grown in eviGrow medium, a chemically defined, animal component-free, serum-free medium. Cells were transfected with eviFect, and cells were grown in eviMake2 after transfection. The supernatant was harvested by centrifugation followed by filtration (0.2 μm filter). The antibody was purified using MabSelect SuRe. Monovalent CD3 binding BTCE was generated using “Knobs-into-holes” technology.

### Animals and treatments

Female GK rats were produced by the mating of male and female GK rats in the conventional Buffon facility (Institut Jacques Monod, Université Paris Cité, Paris, France). All animals were fed ad libitum with a commercial pellet diet (Diet 113; SAFE; Augy, France). They were housed in group (3 adult rats per cage) under specific pathogen-free conditions in a temperature-controlled room (21–22 °C) with a 12 h–12 h light/dark cycle and were weaned at 28 days after birth.

Anovulatory 5–7 months old GK rats were treated by intraperitoneal (IP) injection of 100 μg or 1 mg of rMab22A2, IgG2a (BX-BE0089, Euromedex, Souffelweyersheim, France) or the same volume of PBS according to the protocol described in the Results section. They were sacrificed at the end of the protocols, after an IP injection of domitor (0.25 mg kg^−1^ of body weight) and ketamine (75 mg kg^−1^ of body weight).

### Serum preparation

Blood samples were either taken from the tail for live animals or from the carotid artery after sacrifice. Serum was extracted from the blood by centrifugation (5 min, 1000 *g*, 4 °C) after clotting for 4 h at 4 °C for coagulation and stored at −80 °C until use.

### Determination of AMH, LH, leptin, glucose and insulin concentrations

Serum AMH levels were determined by rat and mouse AMH ELISA (AL-113 (RRID: AB 2783665) AnshLabs, Webster, USA). Serum LH levels were measured by rat LH ELISA (E-EL-R0026; Elabscience, Houston, USA). Serum leptin levels were measured using the mouse/rat leptin Quantikine ELISA (MOB00, R&D, Abingdon, UK). Blood glucose levels were measured using an Accu Check Performa glucometer (Roche, Meylan, France). Serum insulin levels were determined using the Ultrasensitive mouse insulin ELISA, (80-INSRT-E01 (RRID:AB_2792981), ALPCO Diagnostics, Salem, USA).

### Quantification of steroids

Progesterone, testosterone and delta4-androstenedione were determined in rat serum using two-dimensional ultra-performance liquid chromatography-tandem mass spectrometry (2D-UHPLC–MS/MS). For protein precipitation, 30 μL of sample, 20 μL of methanol and 60 μL of a mixture of isotopically labelled internal standards in acetonitrile were added sequentially. The samples were vortexed for 5 s (sec) and centrifuged at 16,000 g for 5 min. Thirty microlitres were injected into the chromatographic system. Chromatographic separation was performed on a Shimadzu Nexera system (Shimadzu France, Marne la Vallée, France) using a two-dimensional method. Sample clean-up and chromatographic separation were performed sequentially using a Shimadzu® MAYI-ODS perfusion column (5 mm L × 2 mm I.D.), and Kinetex Biphenyl (50 × 3 mm I.D, 2.6 μm, Phenomenex, Le Pecq, France). Detection was performed using a triple quadrupole mass spectrometer (LCMS-8060, Shimadzu France). Chromatographic conditions, MRM transitions and method validation on human serum were described in detail in a previous study.^31^ Data were analysed using LabSolutions Insight (Shimadzu France). For quantification, calibration curves were calculated using linear regression with 1/x^2^ weighting.

### Body composition

Body composition was analysed in GK rats using EcoMRI™ 100 (Whole Body Analysers, EchoMRI, Houston, USA). Lean mass and fat mass were determined for each rat. All results are expressed as the percentage of total body weight.

### Oestrous cycle assessment

Oestrous cycle changes were determined as previously described.^19^ Briefly, a glass swab was moistened with physiological serum and inserted into the vagina of rats. The cells were quickly spread on a microscope slide and the smear was left at room temperature until completely dry. The cells were stained with Giemsa (1:10; GS500; Sigma–Aldrich), washed with tap water and examined by light microscopy. The proestrous day was characterised by round nucleated epithelial cells, the oestrous day by a large number of clustered cornified squamous epithelial cells and the metestrous day by the predominance of leucocytes.

### Ovarian histology and follicular number

Right ovaries were fixed in BOUIN solution (HT10132; Sigma) for 24 h at 4 °C. Paraffin-embedded ovaries were sectioned at 5 μm thickness and one of every 10 sections of the ovary was labelled with Proliferating Cell Nuclear Antigen (PCNA) antibody (1:250; M0879 (RRID:AB_2160651) Dako, Santa Clara, USA) as previously described.^19^ Briefly, after antigen retrieval, sections were incubated with 0.3% H_2_O_2_ in pure methanol for 15 min. Non-specific antigenic sites were blocked with 5% horse serum in PBS for 30 min, and the sections were incubated with the mouse anti-PCNA monoclonal antibody overnight at 4 °C. After two PBS washes, the sections were incubated with a biotinylated anti-mouse secondary antibody (1:200; Vectastain Universal Anti Mouse IgG/Rabbit IgG ABC kit, Vector Laboratories, Peterborough, UK) at room temperature for 1 h. After two PBS washes, the slides were incubated with the streptavidin-biotin-peroxidase complex for 30 min. Peroxidase activity was developed using 3,3-diaminobenzidine tetrahydrachloride (Peroxidase Substrate kit, Vector Laboratories). Finally, all sections were counterstained with haematoxylin and the number of follicles was counted under a light microscope using an ×20 objective. Follicles were classified into four classes: primordial follicles with partial or flattened granulosa cells surrounding the oocyte; primary follicles with an oocyte surrounded by a single layer of cuboidal granulosa cells; preantral follicles with 2–6 layers of granulosa cells; and antral follicles with an antral cavitea. Cyst-like follicles showed a thin layer of granulosa cells and a thickened area of the theca cell layer as previously reported.^32^ For the measurement of atretic follicles measurements, antral follicles were considered atretic if they showed pyknotic granulosa cells in the antral cavity and a degenerating oocyte with no connection to the cumulus granulosa cells.^33^ To avoid redundant counting, each follicle was counted only in the section where the nucleolus of the oocyte was visible by comparison with the preceding and following sections. The surface area of each ovarian section was measured using the Histolab software (Microvision Instruments, Evry, France) and the ovarian volume [mm^3^ = Σ (surface area mm^2^ ∗ 5) ∗ 10] was calculated. Follicular count was expressed as follicles per mm^3^ of ovary.

### RNA extraction and RT-qPCR analysis

Left ovaries were harvested, snap frozen in liquid nitrogen and stored at −80 °C until total RNA extraction using the RNeasy Plus Mini kit (Qiagen, Les Ulis, France). 1 μg of RNA was retrotranscribed using the High Capacity cDNA RT kit (Applied Biosystem, Foster City, USA). One-fifth dilutions of the cDNA samples were analysed in duplicate using GoTaq qPCR master mix (Promega) on an QuantStudio1 instrument (ThermoFisher Scientific). For each primer pair (Supplementary Table S1), amplification efficiency was measured using serial dilutions of cDNA. The PCR protocol used an initial denaturation step of 95 °C for 10 min followed by 45 cycles of 95 °C for 10 s and 60 °C for 60 s, followed by melting curve analysis. PCR products for each gene of interest were normalised according to the expression of the housekeeping gene Hypoxantine-Guanine Phosphoribosyltransferase (*Hprt*). Data were obtained as Ct values (Ct = cycle number at which logarithmic PCR plots cross a calculated threshold line) and used to determine ΔCt values [ΔCt = (Ct of the target gene) – (Ct of *Hprt*)]. Results between PBS-treated rats and rMab22A2-treated or IgG2-treated rats were compared using the 2^−ΔΔCt^ method.

### Ethics

All procedures on the rats were performed in accordance with the guidelines of the Council of the European Union (2010/63/UE) and approved by the Institutional Animal Care and Use Ethics Committee of the University Paris-Cité (Agreement B-75-13-17). Animal experiments were also performed in accordance with the ARRIVE (Animal Research: Reporting of In Vivo Experiments) guidelines.^34^ No human participants were involved in this preclinical research study.

### Statistics

Only female rats were used in the experiments and sample size (*n*), P-values, and the types of statistical tests are all indicated in the figures or in the figure legends. Although no statistical methods were used to pre-determine the experimental sample size, we calculated a posteriori using the R language and the pwr library the power of the different statistical tests. The calculated powers were all above 95%, indicating a good ability to detect significant effects with the sample sizes used. The other statistical analyses were carried out using either GraphPad Prism version 10 for Windows (GraphPad Software, La Jolla, California, USA) or the R language for the sign test. The data were first subjected to the Shapiro–Wilk, Kolmogorov–Smirnov and Q–Q plot normality tests, followed by the Fisher and Bartlett tests to assess the homogeneity of variances. Depending on the results, parametric (unpaired or paired t-tests or one-way ANOVA followed by Tukey’s multiple comparison test) or non-parametric (Mann Whitney test, Wilcoxon or sign tests depending on symmetrical distribution, or Kruskal–Wallis test followed by Dunn’s multiple comparison test) tests were used to determine statistical significance. Differences were considered significant when P < 0.05. No data were excluded from analysis. Data are presented as mean values with 95% CI.

### Role of funders

The funders were not involved in the study’s design, data collection, analysis, interpretation, writing or editing of the manuscript.

## Results

### Identification of a monoclonal antibody that blocks the action of AMH, Mab22A2

**Fig. 1:**
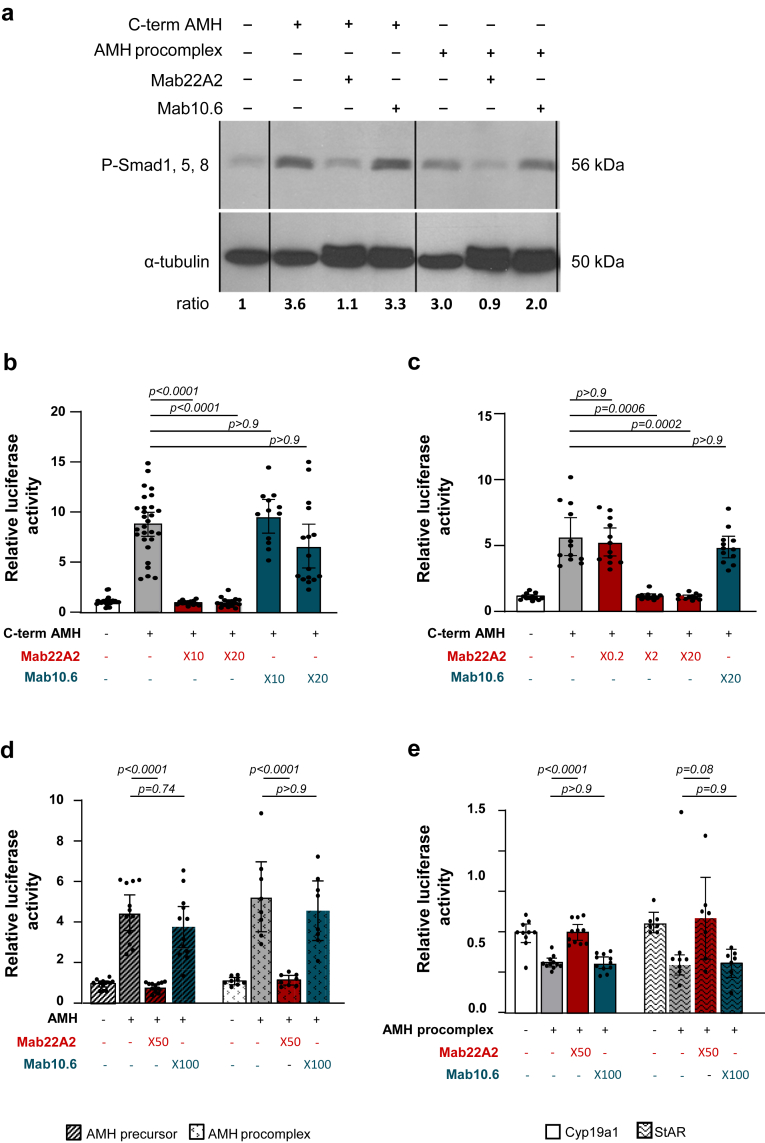
**Mab22A2 neutralises AMH effects in several gonadal cell lines in vitro.** (**a**) Western blot analysis of Smad1, 5, 8 phosphorylation (P-Smad1, 5, 8) in SMAT-1 Sertoli cells, after 1 h (1 h) incubation in control medium or with C-terminal AMH (C-term AMH) (2 nM) or AMH procomplex (7.14 nM) preincubated or not for 1 h with a molar excess of Mab22A2 or Mab10.6 (4 nM or 70 nM of antibody for C-term AMH and AMH procomplex, respectively). The level of P-Smad1, 5, 8 in each sample was calculated from a densitometric analysis of the bands corresponding to P-Smad1, 5, 8 and α-tubulin used as gel loading control. The fold increase of P-Smad1, 5, 8 compared to control medium is shown at the bottom of each lane. (**b**) SMAT-1 cells were co-transfected with the Smad5 reporter system and pRLTK plasmid as a control for transfection efficiency. Luciferase activity was assessed after 24 h of treatment with either control medium or C-term AMH (2 nM) pre-incubated or not 1 h with an excess of Mab22A2 or Mab10.6 (20 and 40 nM of each antibody). (**c** and **d**) KK1 granulosa cells were co-transfected with the Smad5 reporter system, mouse AMHR2 cDNA and pRLTK plasmid. Luciferase activity was assessed after 24 h of treatment with either control medium, (**c**) C-term AMH (2 nM) pre-incubated for 1 h or not with increasing excess of Mab22A2 (0.4, 4 and 40 nM) or Mab10.6 (40 nM) (**d**) AMH precursor (7.14 nM) or AMH procomplex (7.14 nM) pre-incubated with or without an excess of Mab22A2 (350 nM) or Mab10.6 (700 nM) for 1 h. (**e**) KK1 cells were co-transfected with mouse AMHR2 cDNA, pRLTK plasmid and either an Cyp19a1 or a StAR reporter gene. Luciferase activity was assessed after 24 h of treatment with either control medium, or AMH procomplex (7.14 nM) pre-incubated or not for 1 h with an excess of Mab22A2 (350 nM) or Mab10.6 (700 nM). Results were expressed as the ratio of Firefly to Renilla luciferase activity. Each point represents a replicate of at least 3 experiments. Data are presented as a scatter plot bar with mean with 95% CI. P-values are from Kruskal–Wallis test followed by Dunn’s multiple comparison test (b, c, d) or one-way ANOVA test followed by Tukey’s multiple comparison test (e).

Several anti-human AMH monoclonal antibodies have previously been prepared with the aim of setting up an ELISA.^24^ We used Western-blotting to test whether one of them, Mab22A2, which recognises the C-terminal bioactive domain of AMH, was able to block AMH signalling in an AMH-responsive cell line, mouse SMAT-1 Sertoli cells.^35^ Incubation of either human C-terminal AMH or AMH procomplex with a molar excess of Mab22A2 for 1 h, blocked the phosphorylation of Smad1,5, 8 induced by these AMH samples (Fig. 1a). In contrast, Mab10.6, which recognises the N-terminal AMH pro-domain, did not prevent AMH-stimulated phosphorylation. To confirm the blocking effect of Mab22A2 using a different approach, we then transfected SMAT-1 cells with a Smad5 reporter system consisting of a Gal4-Smad5 fusion protein and a Gal4-luciferase reporter construct.^25^ Mab22A2 blocked the stimulation of luciferase activity by C-terminal AMH whereas Mab10.6 had no effect on luciferase induction (Fig. 1b). We next wanted to investigate whether Mab22A2 could block AMH signalling in ovarian AMH target cells, such as granulosa cells. We used the mouse granulosa cell line KK1, which responds to AMH after transfection with mouse AMHR2 cDNA (Supplementary Fig. S1a). After co-transfection of the Smad5 reporter system in these cells, we found that a molar excess of Mab22A2 prevented the induction of luciferase by C-terminal AMH (Fig. 1c). In addition, Mab22A2 also blocked the effect of the different circulating forms of AMH, AMH precursor and AMH procomplex, on the transcriptional activities of the Smad5 (Fig. 1d) and Smad1 promoters (Supplementary Fig. S1b). Finally, we tested whether Mab22A2 could block the effects of AMH on two of its known target genes: Cyp19a1 (aromatase gene) and steroidogenic acute regulatory protein (StAR, which regulates cholesterol transport within the mitochondria) genes, as observed in granulosa cells.^36^^,^^37^ As shown in Fig. 1e, the AMH procomplex reduced the luciferase activity of the Cyp19a1 and StAR reporter genes in KK1 cells by 36.7% and 46.7% respectively; Mab22A2 blocked these effects, whereas Mab10.6 did not.

### Mab22A2 does not prevent AMH binding to its receptor and is interspecific

**Fig. 2:**
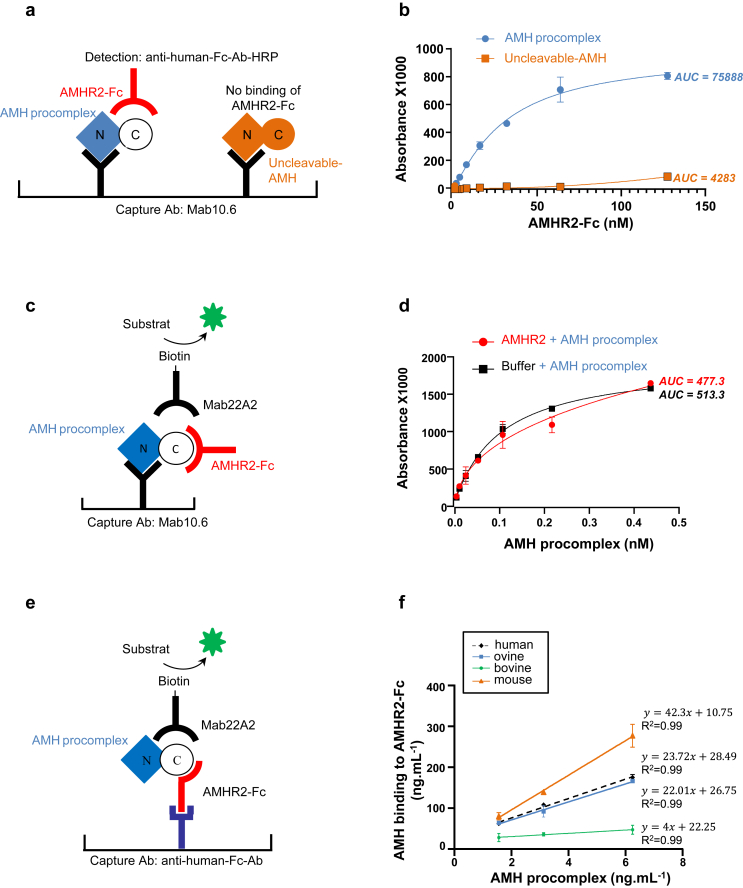
**Characterisation of Mab22A2.** (**a** and **b**) Strategy to study the effect of Mab22A2 on the interaction between AMH and its type two receptor AMHR2. (**a**) ELISA format and (**b**) results showing binding of AMH procomplex but not uncleavable AMH to AMHR2. AMH was captured on Mab10.6 and incubated with various concentrations of a soluble fusion protein containing the extracellular domain of human AMHR2 and the fragment crystallisable region (Fc) portion of human IgG1 (AMHR2-Fc); bound AMHR2-Fc was detected with an anti-human Fc antibody (Ab) conjugated to horseradish peroxidase (HRP). (**c**) ELISA format and (**d**) results showing that Mab22A2 recognises AMH procomplex without preventing its interaction with AMHR2. Different concentrations of AMH procomplex were captured on Mab10.6 and incubated with AMHR2-Fc; bound AMH procomplex was detected with biotinylated Mab22A2 and streptavidin-HRP conjugate. The means areas under the curves (AUC) are indicated next to the curves (b, d). (**e**) ELISA format and (**f**) results showing that Mab22A2 recognises AMH from different species. AMHR2-Fc was captured with a goat α-human Fc Ab and incubated with human recombinant AMH procomplex or AMH procomplex present in bovine, ovine and mouse sera, at three different dilutions. Bound AMH was detected with biotinylated Mab22A2 and streptavidin-HRP conjugate. The slopes and the correlation coefficients for the AMH procomplex and the three serum samples are given next to the different straight lines.

We then investigated how Mab22A2 blocks the effect of AMH. To do this, we used an ELISA format validated in Fig. 2a and b and in.^23^ Two different forms of AMH, AMH procomplex and uncleavable AMH which contains the R451T mutation at the monobasic cleavage sites, making it resistant to cleavage and biologically inactive, were captured by Mab10.6 coated on the plates. A soluble fusion protein containing the extracellular domain of human AMHR2 and the Fc portion of human IgG1, AMHR2-Fc was then added, and bound AMHR2-Fc was detected with an anti-human Fc secondary antibody.^23^ As shown in Fig. 2b, the AMHR2-Fc fusion protein could only bind to the bioactive AMH procomplex (AMH procomplex: mean AUC = 75,888, upper 95% CI = 90,463, lower 95% CI = 61,314; uncleavable AMH: mean AUC = 4283, upper 95% CI = 6963, lower 95% CI = 1604, n = 3). When biotinylated Mab22A2 was used to detect the AMH procomplex, the signal was not affected by a pre-incubation step with AMHR2-Fc (Fig. 2c and d) (buffer: mean AUC = 477.2 upper 95% CI = 573.9, lower 95% CI = 380.6; AMHR2-Fc: mean AUC = 513.3, upper 95% CI = 532.9, lower 95% CI = 493.7, n = 3). This result indicates that Mab22A2 and AMHR2-Fc bind to different epitopes on the C-terminal AMH domain and that Mab22A2 blocks AMH signalling without preventing its binding to AMHR2, but rather by inhibiting either AMHR2 dimerisation or type I receptor activation. To assess the interspecificity of Mab22A2, we then used another ELISA format, in which the AMHR2-Fc fusion protein was captured by an anti-human Fc secondary antibody coated on the plate (Fig. 2e). After incubation with sera from three different species, the amount of bound AMH procomplex was detected by biotinylated Mab22A2. Fig. 2f shows that, in addition to human recombinant AMH procomplex, Mab22A2 recognises bovine, ovine and mouse AMH procomplex present in the serum, demonstrating that it is interspecific, and allowing its use for pre-clinical studies.

### Treatment of GK rats with rMab22A2

#### Preparation of Mab22A2 in a rat format (rMab22A2) and selection of anovulatory GK rats

Next, we wanted to assess the ability of Mab22A2 to block AMH in vivo. For this purpose, we took advantage of GK rats, which we have shown to have the main features of PCOS.^19^ Indeed, these rats have elevated serum levels of AMH and testosterone, which could be involved in their ovulation defects from 3.5 months of age, and in the blockade of oestrous cycles at the proestrus (PE)/oestrus (E) stage at 6 months of age in 75% of GK rats. To test whether Mab22A2 treatment could alleviate any of these symptoms, anovulatory GK rats (5–7 months old) with oestrous cycles blocked at the PE/E cycle for 15 days (corresponding to 3 oestrus cycles) were selected by daily analysis of their vaginal smears. In parallel, to avoid a possible immunogenic reaction associated with repeated injections, a rat version of Mab22A2, rMab22A2, was produced: the variable regions of Mab22A2 (IgG1a) were cloned and sequenced by the Biotem company (Table 1). They were then inserted into a rat IgG2c backbone by Evitria, N234A, L234A and P329G substitutions were introduced into the Fc to reduce Fc receptor interactions, and expression of rMab22A2 was carried out in CHO-K1 cells.

**Table 1:** Nucleotide and protein sequences encoding the variable domains of the Mab22A2 light and heavy chains.

a Sequence encoding the variable domain of the Mab22A2 light chain (VL) 1. Nucleotide sequence VLᴋ GACATCCAGATGACTCAGTCTCCAGACTCCCTATCTGCATCTGTGGGAGAAACTGTCACCATCACATGTCGAGCAAGTGAAAATATTCAGAACTATTTAGCATGGTATCAGCATAAACAGGGAAAATCTCCTCAGCTCCTGGTCTATAATGCACAAACCTTAGCGAATGGTGTGTCATCAAGGTTCAGTGGCAGTGGATCAGGAACACATTATTCTCTCAATATCCATAGTCTGCAGCCTGAAGATTTTGGGACTTATTACTGTCAACATTTTTGGGGTACCCCTCGGACGTTCGGTGGAGGCACCAAACTAATAATCAAACGGGCTGATGCTGCACCAACTGTATCCATCTTCCCACCATCC 2. Protein sequence VLᴋ DIQMTQSPDSLSASVGETVTITCRASENIQNYLAWYQHKQGKSPQLLVYNAQTLANGVSSRFSGSGSGTHYSLNIHSLQPEDFGTYYCQHFWGTPRTFGGGTKLIIKRADAAPTVSIFPPS Partial sequences of the constant domain of Ck (Mus musculus IGKC∗01).
b Sequence encoding the variable domain of the Mab22A2 heavy chain (VH) 1. Nucleotide sequence VH CAGATCCGATTGGTGCAGTCTGGACCTGAACTGAAGAAGCCTGGAGAGACAGTCAAGATCTCCTGTAAGGCTTCTGGATATTCCTTCACAAACTATTTAATGCACTGGGTGAAACAGGCTCCAGGAGAGGCTTTAAAGTGGATGGGCTGGATAAACACCAACATTGGACAGCCAACATATGCTGAAGATTTCAAGGGACGGTTTGTCTTCTCTTTGGAAGCCTCTATCAGTACTGCCTTCTTACACATCAACAGTCTCAAAGATGAAGACACGGCTACATATTTCTGTACAAGTGGTTACTTCGGCTACGACTGGTACTTCGATGTCTGGGGCGTAGGGACCACGGTCACCGTCTCCTCAGCCAAAACGACACCCCCATCTGTCTATCCACTGGCCCCTGGATCTGCTGCCCAAACTAACTCCATGGTG 2. Protein sequence VH QIRLVQSGPELKKPGETVKISCKASGYSFTNYLMHWVKQAPGEALKWMGWINTNIGQPTYAEDFKGRFVFSLEASISTAFLHINSLKDEDTATYFCTSGYFGYDWYFDVWGVGTTVTVSSAKTTPPSVYPLAPGSAAQTNSMV Partial sequences of the constant domain CH1 (Mus musculus IGHG1∗01)

Sequencing of the variable regions of Mab22A2 was performed by the Biotem company. (**a**) Sequence encoding the variable domain of the kappa light chain (VLκ): 1. Nucleotide sequence, 2. Protein sequence. The partial sequences of the constant domain of Ck (Mus musculus IGKC∗01) are shown in red. (**b**) Sequence encoding the variable domain of the heavy chain (VH): 1. Nucleotide sequence, 2. Protein sequence. The partial sequences of the constant domain CH1 (Mus musculus IGHG1∗01) are indicated in red.

#### Treatment of anovulatory GK rats with a single acute injection of 1 mg of rMab22A2

**Fig. 3:**
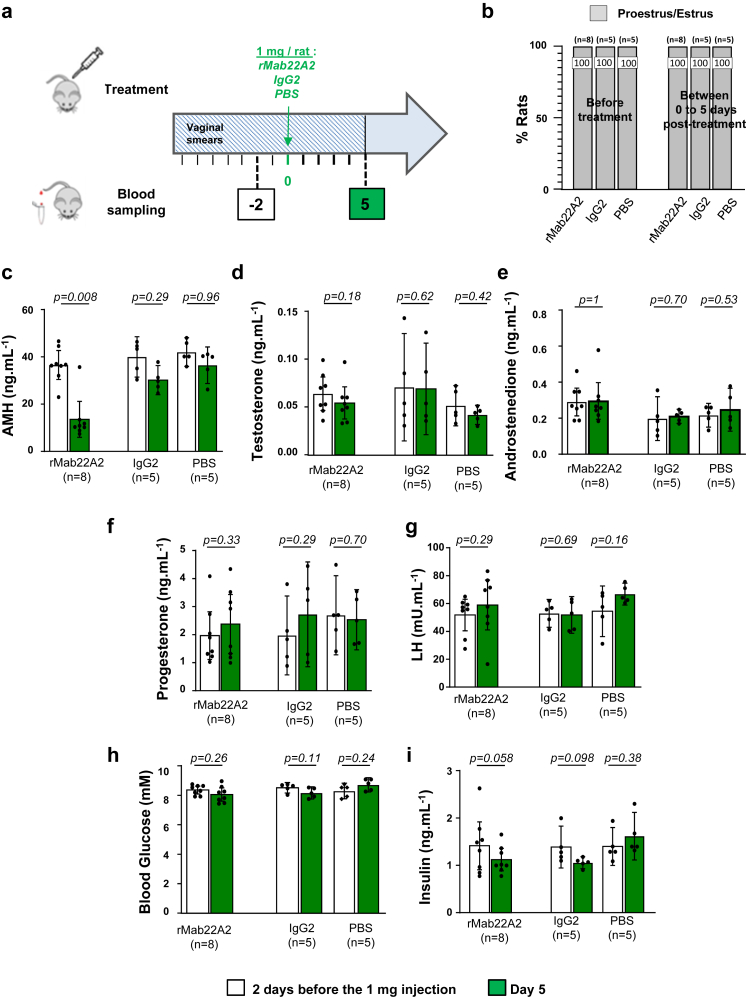
**rMab22A2 treatment with a single acute dose reduces bioavailable AMH levels in GK rats in vivo.** (**a**) Diagram showing the protocol with a single acute injection of 1 mg of rMab22A2, IgG2 or PBS. The anovulatory status of 5–7 months old GK rats was checked by daily analysis of their vaginal smears for 15 days prior to treatment with a single intraperitoneal (IP) injection of 1 mg of rMab22A2, control IgG2 or PBS. Oestrous cycles were monitored daily from the IP injection, and until 5 days after this acute treatment. Blood samples were taken from the tail of live animals two days before, and five days after this treatment (Day 5). (**b**) Results of oestrous cycle analysis from Day 0 to Day 5. (**c, d, e, f, g h and i**) Serum levels of anti-Müllerian hormone (AMH) (**c**), testosterone (**d**), androstenedione (**e**), progesterone (**f**), luteinising hormone (LH) (**g**), glucose (**h**) and insulin (**i**). Data are presented as a scatter plot bar (white bars: samples collected 2 days before treatment, and green bars: samples collected on Day 5), with mean values with 95% CI. Each point is representative of one rat and *n* represents the number of animals in each group. P-values are from two-tailed t-test or sign test (rMab22A2 group in c, e, g) for paired comparisons.

The first treatment regimen tested was an acute IP injection of 1 mg of the rMab22A2 (n = 8), a classical dose for immunotherapy in rats.^38^^,^^39^ Two other batches of animals (n = 5 each) were treated with a control IgG2 antibody or with PBS, the vehicle of rMab22A2. The oestrous cycles of the treated rats were monitored for 5 days after injection by daily analysis of their vaginal smears (Fig. 3a). Serum samples were collected 2 days before, and 5 days after the 1 mg injection (Day 5); for ethical reasons, only 150 μL of serum can be collected once a week. The oestrous cycle of GK rats treated with rMab22A2 remained blocked at the PE/E stage throughout the period analysed (Fig. 3b), indicating that this protocol did not unblock ovulation. This antibody treatment caused a 63% reduction in detectable serum AMH levels by ELISA (Day 5, Fig. 3c), suggesting that rMab22A2 had sequestered some of circulating AMH, and that detectable AMH probably corresponds to bioavailable AMH. The levels of the other reproductive hormones (testosterone, androstenedione, progesterone and LH) and of some metabolic parameters (blood glucose and insulin levels) were not different before and after rMab22A2 treatment (Fig. 3d–i). IgG2 or PBS treatment also had no effect on any of the hormones (Fig. 3c–i). Taken together these results indicated that treatment with a single acute injection of 1 mg of rMab22A2 did not unlock the oestrous cycle of GK rats but induced a decrease in bioavailable AMH levels in anovulatory GK rats, suggesting that this antibody could neutralise the effects of AMH in vivo.

#### Treatment of anovulatory GK rats with eight chronic injections of 100 μg of rMab22A2

**Fig. 4:**
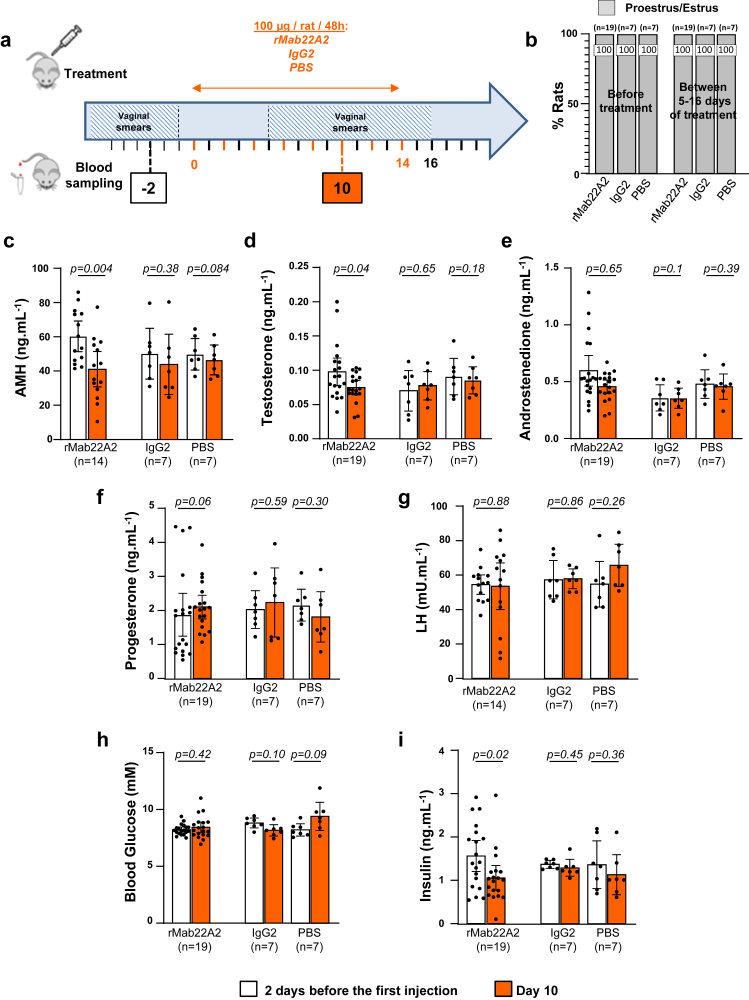
**Chronic low-doses treatment with rMab22A2 reduces the testosterone and insulin levels in GK rats.** (**a**) Diagram showing the protocol with eight chronic injections of 100 μg of the rMab22A2, IgG2 or PBS. The anovulatory status of 5–7 months old GK rats was checked by daily analysis of their vaginal smears during 15 days before they were treated with 8 intraperitoneal (IP) injections of 100 μg of rMab22A2, control IgG2 or PBS every 2 days. Oestrous cycles were monitored daily from day 6 after the start of the IP injections, and until 2 days after the end of this chronic treatment. Blood samples were taken from the tail of live animals two days before (white bars) and 10 days after the first injection (Day 10, orange bars). (**b**) Results of oestrous cycle analysis from Day 5 to Day 16. (**c, d, e, f, g, h** and **i**) Serum levels of anti-Müllerian hormone (AMH) (**c**), testosterone (**d**), androstenedione (**e**), progesterone (**f**), luteinising hormone (LH) (**g**), glucose (**h**) and insulin (**i**) 2 days before (white bars), and on Day 10 (orange bars). Data are represented as a scatter plot bar, with mean values with 95% CI. Each point is representative of one rat and *n* represents the number of animals in each group. P-values are from two-tailed t-test or sign test (rMab22A2 group in e, f, i) for paired comparisons.

We speculated that the normalisation of oestrous cycles in GK rats might require a longer exposure to rMab22A2 due to their prolonged reproductive senescence. Therefore, we next investigated whether treating anovulatory GK rats with almost the same total amount of rMab22A2 (800 μg), but over a period of 14 days (Fig. 4a) could improve their reproductive parameters better than a single acute injection. Other batches of anovulatory GK rats were therefore treated over 14 days, with 8 IP injections of 100 μg of rMab22A2, IgG2 or PBS every 48 h. Oestrous cycles were assessed daily from 6 days after the first IP injection, and until 2 days after the end of this chronic treatment. Serum samples were collected 2 days before, and 10 days after the first injection (Day 10). Similar to the results of the first protocol (the single acute injection, Fig. 3), anovulatory GK rats treated with rMab22A2 stayed blocked at the PE/E stage throughout the period analysed (Fig. 4b). As shown in Fig. 4c, this protocol reduced bioavailable AMH levels at Day 10, but to a lesser extent than the single 1 mg injection (31.8% vs. 63% (Fig. 3c)), and to levels still higher than Wistar rats (41.07 ± 4.8 vs. 21.5 ± 2.54 ng mL^−1^ for Wistar rats^19^). In addition, rMab22A2 treatment also decreased both testosterone and insulin concentrations by 23.7% and 32.4% respectively (Fig. 4d and i), to levels close to Wistar rats for testosterone (0.075 ± 0.005 vs. 0.06 ± 0.02 ng mL^−1^ for Wistar rats^19^), and lower than Wistar rats for insulin (1.05 ± 0.14 vs. 1.56 ± 0.36 ng mL^−1^ for Wistar rats^19^). In contrast, this treatment had no effect on androstenedione, progesterone, LH, glucose and leptin levels, or on adipose tissue repartition measured by EcoMRI (Fig. 4e–h and Supplementary Fig. S2). Treatment of anovulatory GK rats with either IgG2 or PBS had no significant effect on reproductive or metabolic parameters (Fig. 4c–i). Taken together, these results showed that treatment of anovulatory GK rats with 8 injections of 100 μg of rMab22A2, did not unlock the oestrous cycle of GK rats but reduced their bioavailable AMH levels, and also their serum androgen and insulin concentrations.

#### Treatment of anovulatory GK rats with eight injections of 100 μg followed by one acute injection of 1 mg of rMab22A2

**Fig. 5:**
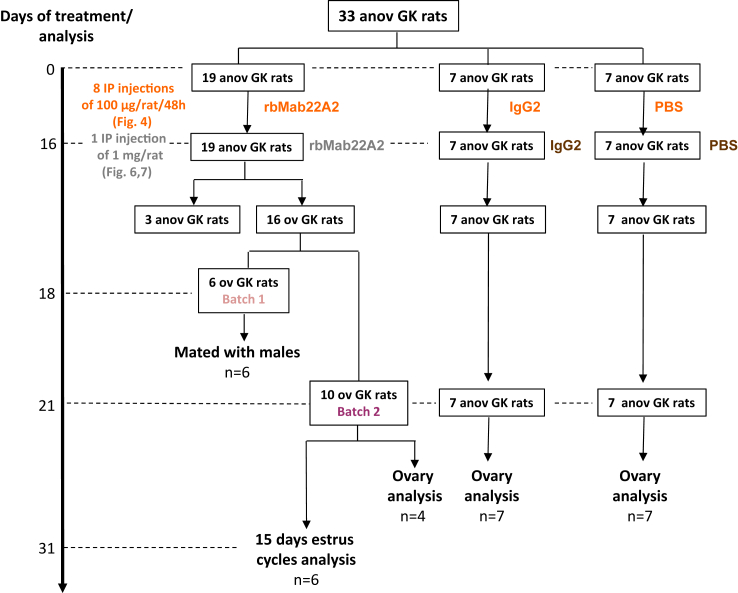
**Flowchart of the treatments and experiments performed in**Fig. 4, Fig. 6, Fig. 7**.** Anovulatory (anov) 5–7 month old GK rats (n = 33) were treated with 8 intraperitoneal (IP) injections of 100 μg of rMab22A2 (n = 19), control IgG2 (n = 7) or PBS (n = 7) every 2 days (Fig. 4), followed 2 days after the last injection, on Day 16 from the start of the treatment, by a single IP injection of 1 mg of rMab22A2, control IgG2 or PBS (Fig. 6, Fig. 7). Of the 19 GK rats treated with rMab22A2, 16 had ovulated (ov) on Day 18 from the beginning of the treatment (Fig. 6b). Of the 16 GK rats that had ovulated, 6 GK rats (Batch 1) were mated with GK males (Fig. 6c). The other 10 GK rats that had ovulated (Batch 2), were either sacrificed on Day 21 to analyse their ovaries (n = 4) (Fig. 6d, e, f), or studied until Day 31 to assess their oestrous cycles (n = 6) (Fig. 6b). IgG2-treated rats were sacrificed on Day 21 to collect serum samples (n = 7) (Fig. 7b–h) and/or to analyse their ovaries (n = 7) (Fig. 6f). PBS-treated rats were sacrificed on Day 21 to collect serum samples (n = 7) (Fig. 7b–h) and/or to analyse their ovaries (n = 4) (Fig. 6d, e) and (n = 7) (Fig. 6f). Blood samples were collected from the tail two days before and at Day 10 for all the rats (n = 33) (Fig. 4), at Day 18 for Batch 1 ov GK rats treated with rMab22A2 before mating with males (n = 6) (Fig. 7b–h), and on Day 21 for all Batch 2 ov GK rats treated with rMab22A2 (n = 10), and anov GK rats treated with either IgG2 or PBS (n = 7 each) (Fig. 7b–h).

**Fig. 6:**
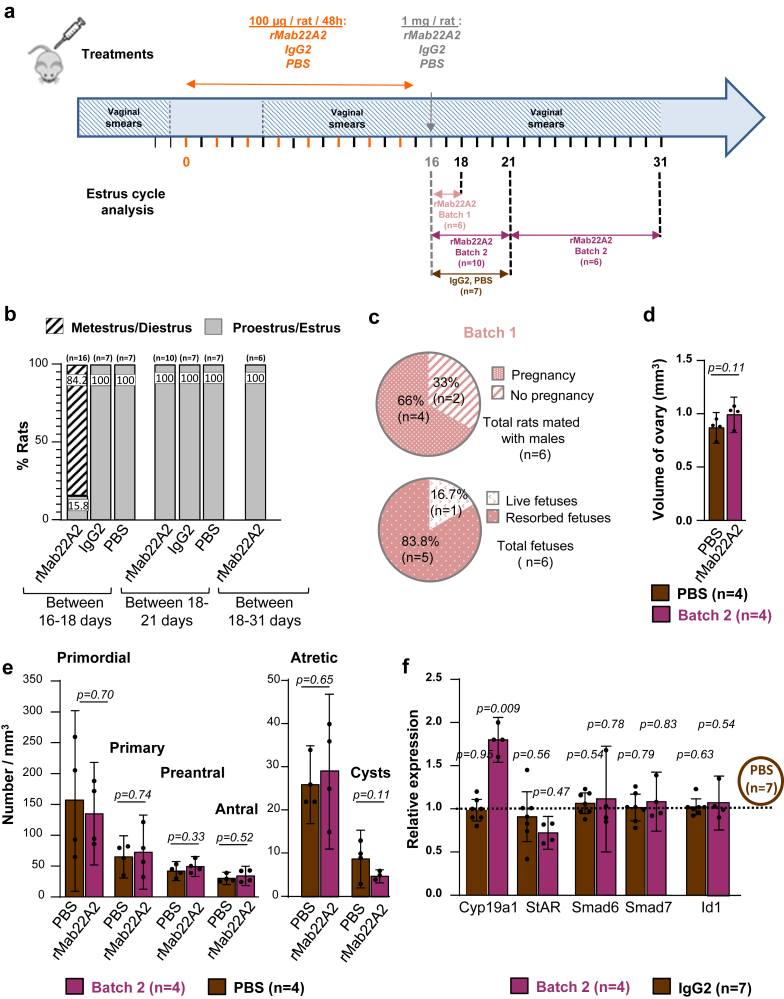
**rMab22A treatment with the combined chronic and acute protocols unblocks the oestrous cycle in GK rats.** (**a**) Diagram showing the protocol with eight injections of 100 μg followed by an acute injection of 1 mg of rMab22A2, IgG2 or PBS. The same GK rats treated with 8 intraperitoneal (IP) injections of 100 μg of rMab22A2, control IgG2 or PBS every 2 days (Fig. 4), were treated 2 days after the last injection, with a single IP injection of 1 mg of rMab22A2, control IgG2 or PBS. Oestrous cycles were monitored daily from Day 6 after the start of the IP injections, and until Days 18, 21 and 31 after. On Day 18, Mab22A2-treated rats were divided into two batches: Batch 1 rats (n = 6) were mated with GK males on Day 18, and Batch 2 rats (n = 10) were subjected to further analyses from Day 18 to Day 31. IgG2- or PBS-treated rats were sacrificed on Day 21. (**b**) Results of oestrous cycle analysis from Day 16 to Day 31. (**c**) Percentage of pregnancies and foetuses for Batch 1 rats treated with rMab22A2. (**d**) Determination of the ovarian volume for a subset of Batch 2 rats (n = 4, purple bars) and PBS-treated rats (n = 4, brown bars) on Day 21. (**e**) Follicle content for part of Batch 2 rats (n = 4, purple bars) and PBS-treated rats (n = 4, brown bars) on Day 21. (**f**) Expression of AMH-target genes in Batch 2 rats (n = 4, purple bars) and in IgG2-treated rats (n = 7, brown bars) by RT-qPCR relative to PBS-treated rats (n = 7), defined as 1 (hatched horizontal line). Data are represented as a scatter plot bar, with mean values with 95% CI. Each point is representative of one rat and *n* represents the number of animals in each group. P-values are from unpaired t-test or Mann–Whitney test (in f, StAR).

To further increase the exposure of anovulatory rats to rMab22A2, we tested whether a combination of chronic and acute injections could unblock their oestrous cycles. To this end, the same rats treated with 8 injections of 100 μg of antibody (Fig. 4), received an IP injection of 1 mg of the corresponding antibodies, 48 h after the last injection of 100 μg of antibody, on Day 16 (Fig. 5, Fig. 6a). Two days after this acute injection, on Day 18, 16 out of 19 rats (84.2%) treated with rMab22A2 were in metoestrus (Fig. 6b), indicating that they had ovulated, and that this protocol had unlocked their oestrous cycle. In contrast, 100% of GK rats injected with IgG2 or PBS remained blocked at the PE/E stage of their oestrous cycle. The rMab22A2-treated rats that ovulated (n = 16) were then divided into two batches (Fig. 5): six rats were mated with GK males on Day 18 (Batch 1), while 10 rats were used for other analyses (Batch 2). Four Batch 2 rats were sacrificed on Day 21 for ovarian analysis and six Batch 2 rats were analysed up to Day 31 for oestrus cycle assessment. IgG2 or PBS treated rats were sacrificed on Day 21 for ovarian analysis and/or serum sampling.

Mating of the six Batch 1 rats to GK males resulted in 4 pregnancies, representing a percentage of 66% of pregnancies (Fig. 6c) (compared to 50% in 5 months old Wistar rats, n = 18, Supplementary Table S2). The 4 pregnant rats were euthanised at 18.5 days of gestation in order to count their foetuses as GK rats do not care for their pups, and there is potential loss of offspring after birth. Out of a total of 6 foetuses, one male was alive and 5 were aborted (Fig. 6c). Indeed, GK rats already have very small litters and an increased number of aborted foetuses at 3 months,^19^ a phenomenon that is likely to be amplified at 6 months. Analysis of the oestrous cycles of Batch 2 rats showed that from Day 18 to Day 31, GK rats remained in the PE/E stage (Fig. 6b), meaning that their oestrous cycles were blocked again, and that they had ovulated only once. The analysis of the volume (Fig. 6d) and of follicle content (Fig. 6e) of GK rat right ovaries at Day 21 did not reveal any difference between GK rats treated with rMab22A2 and those treated with PBS, although the number of cysts tended to be lower in the rMab22A2-treated rats.

We also studied the expression of several AMH target genes in the left ovaries of Batch 2 rats and of PBS- and IgG2-treated rats sacrificed on Day 21 (Fig. 6f). We observed no effect of rMab22A2 treatment on the expression of the early AMH-target genes Smad6, Smad7 and Id1,^9^ and of StAR, compared to PBS treatment. In contrast, the expression of Cyp19a1 was increased in rMab22A2-treated rats by 81.2%, indicating that this treatment had regulated ovarian steroidogenesis in GK rats. IgG2 treatment had no effect on the expression of any of these genes compared to PBS treatment.

**Fig. 7:**
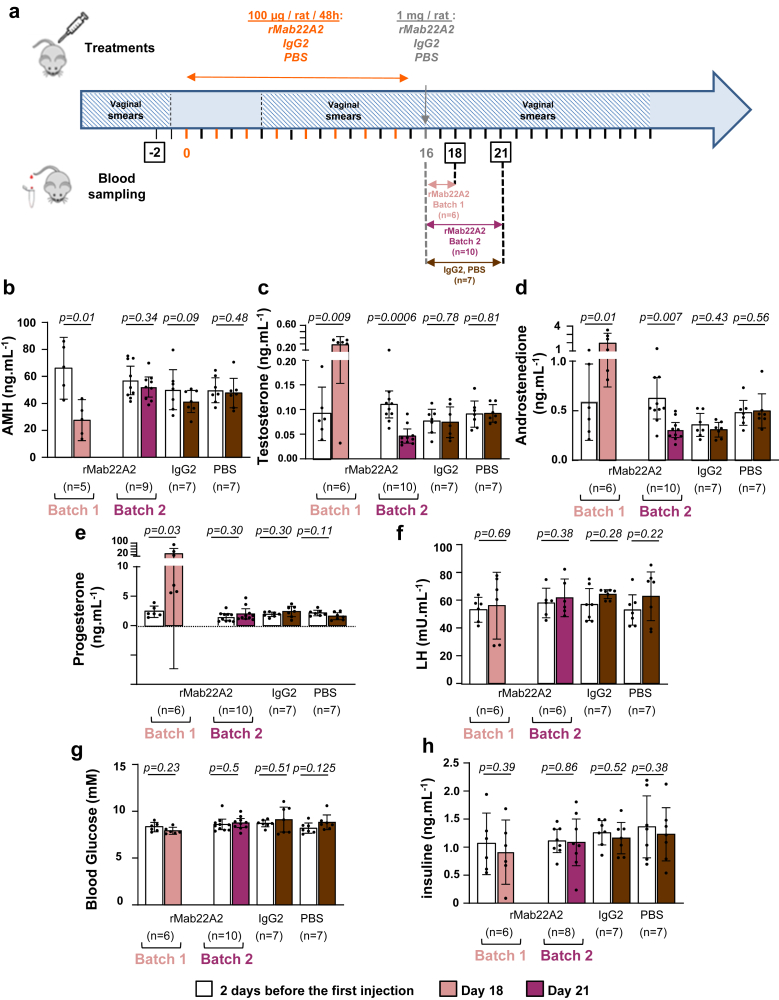
**rMab22A treatment with the combined chronic and acute protocols normalises both bioavailable AMH concentrations and androgen levels.** (**a**) Diagram showing the protocol with eight injections of 100 μg followed by an acute injection of 1 mg of rMab22A2, IgG2 or PBS. Blood samples were collected from the tail of live animals or at sacrifice on Days 18 and 21 after the start of the treatment. (**b, c, d, e, f, g** and **h**) Serum levels of anti-Müllerian hormone (AMH) (**b**), testosterone (**c**), androstenedione (**d**), progesterone (**e**), luteinising hormone (LH) (**f**), glucose (**g**) and insulin (**h**) 2 days before treatment (white bars), and on Day 18 (salmon bars for Batch 1 rats) and Day 21 (purple bars for Batch 2 rats, brown bars for IgG2- or PBS-treated rats) after treatment. Data are represented as a scatter plot bar, with mean values with 95% CI. Each point is representative of one rat and *n* represents the number of animals in each group. P-values are from two-tailed t-test or Wilcoxon test (rMab22A2 groups in e, IgG2 group in f) or sign test (PBS group in g) for paired comparisons.

To assess the effects of the combined protocol on reproductive and metabolic markers, serum samples were collected on Day 18 prior to mating for Batch 1 rats, and on Day 21 for Batch 2 rats, and for control rats treated with IgG2 or PBS. On Day 18, Batch 1 rats had significantly lower bioavailable AMH levels compared to pre-treatment (58.4% reduction), which were similar to those of Wistar rats (27.56 ± 5.5 vs. 21.5 ± 2.54 ng mL^−1^ for Wistar rats^19^). Batch 2 rats had no significant reduction in AMH levels on Day 21 (5 days after the end of the treatment) compared to pre-treatment levels (Fig. 7b). The reason why bioavailable AMH levels increased between Day 18 and Day 21, i.e., 2–5 days after the last rMab22A2 injection although the half-life of monoclonal antibodies is about 3 weeks, deserves further investigation; this could be due to the fact that the serum samples on Day 18 (Batch 1) and on Day 21 (Batch 2) were not taken from the same rats, to the degradation of rMab22A2, and/or to the constant production of AMH by the GK ovaries. On Day 18, testosterone, androstenedione and progesterone levels were elevated in Batch 1 rats, consistent with the rats having ovulated during this period of time (progesterone is the precursor of androgens). By Day 21, progesterone of Batch 2 rats had returned to levels observed before the treatment, in keeping with the blockade of their oestrous cycle at PE/E stage from Day 18. In contrast, testosterone and androstenedione levels were lower on Day 21 than pre-treatment levels (58.2% and 50.4% reduction for testosterone and androstenedione, respectively), with testosterone levels similar to those of Wistar rats^19^ (0.06 ± 0.02 ng mL^−1^), but androstenedione concentrations still elevated compared to Wistar rats^19^ (0.06 ± 0.03 ng mL^−1^). No changes were observed in LH, blood glucose, and insulin levels on Days 18 or 21 compared to pre-treatment levels (Fig. 7f–h). Treatment of anovulatory GK rats with IgG2 or PBS had no effect on any reproductive or metabolic parameters (Fig. 7b–h).

Overall, these results showed that treatment of anovulatory GK rats with rMab22A2 according to this third protocol, reduced both their bioavailable AMH concentrations and their androgen levels, but also restored an oestrous cycle in 84% of anovulatory GK rats, as evidenced by the analysis of their vaginal smears, their high progesterone levels 2 days after the end of treatment and the presence of 4 pregnancies among the 6 rats mated with males.

## Discussion

In this article, we report the identification of a blocking antibody against AMH, Mab22A2, which neutralises the effects of AMH both in vitro and in vivo. Indeed, we have shown that this antibody is able to block well characterised effects of AMH in granulosa cells: namely its ability to activate the Smad 1, 5, 8 pathway and to inhibit the transcription of the steroidogenic genes Cyp19a1 and StAR. We have also shown that injection of a rat version of this antibody, rMab22A2, reduces bioavailable serum AMH levels in the anovulatory GK rat, a spontaneous model of PCOS that displays all the features of women with the syndrome. Finally, we showed that treatment of anovulatory GK rats with rMab22A2 also reduced the androgen levels and unlocked the oestrous cycle of anovulatory GK rats, counteracting two putative effects of AMH in the PCOS.

The clinical utility of AMH in gynaecology began to be evaluated in the 2000s. Serum AMH levels were first identified as an early marker for the diagnosis and follow-up of granulosa cells tumours.^24^ In reproductive medicine, serum AMH was recognised as a reliable marker of the ovarian follicular status and a predictor of the ovarian response to controlled ovarian stimulation.^40^^,^^41^ More recently, it has been suggested that AMH treatment administered prior to controlled ovarian stimulation with gonadotropins could improve oocyte yield and synchronisation of follicular development.^42^ In the field of oncofertility, AMH treatment may also prove useful in preventing follicular loss observed during chemotherapy treatment, or an ovarian transplantation (reviewed in^43^^,^^44^). In addition, AMH treatment could be used as a contraceptive (reviewed in^45^ and^46^). In contrast, to our knowledge, no blocking antibody against AMH, AMHR2, or any other strategy to block AMH effects in the context of the PCOS has been developed so far. In fact, blocking antibodies against AMH^47^ or AMHR2^48^^,^^49^ have only been tested to prevent the proliferation of ovarian cancer cells.

The effects of AMH on aromatase expression and androgen production on the one hand, and on terminal folliculogenesis, i.e., dominant follicle selection and ovulation on the other hand, have long been documented in a physiological context (reviewed in^4^). Here, the fact that rMab22A2 treatment reduced androgen levels in GK rats (second and third protocols, Fig. 4, Fig. 7), clearly demonstrated that their high AMH levels contributed to their hyperandrogenism. Consistent with a direct inhibitory effect of high AMH levels on the aromatase activity of granulosa cells, Cyp19a1 expression was increased in rMab22A2-treated ovaries (Fig. 6f). Rat Mab22A2 may also counteract the stimulatory effect of AMH on GnRH neurons and LH production,^10^ although LH levels did not seem to change during the treatment (Fig. 7f). The absence of effect of rMab22A2 treatment on expression of StAR may be due to the fact that this protein is expressed by both granulosa and theca cells. The ovulation of GK rats within 2 days after the acute injection of rMab22A2 in the third protocol (Fig. 6), which corresponds to the time required for antral follicles to grow into preovulatory follicles, also proved that the high AMH levels in GK rats inhibited their terminal folliculogenesis and were involved in their ovulatory defects.

Although it was not possible to determine the exact kinetics of bioavailable AMH and androgen levels in the same rats, our hypothesis is that normalisation of both hormone concentrations between Day 10 and Day 18, allowed ovulation in GK rats treated with rMab22A2. Indeed, in these rats, the bioavailable AMH levels were reduced on Day 10 (Fig. 4), and were comparable to those of Wistar rats on Day 18 (Fig. 7). Testosterone concentrations in rMab22A2-treated GK rats were also reduced to levels close to those of Wistar rats on Day 10 (Fig. 4) and Day 21 (Fig. 7), when the rats were at the same PE/E stage of their oestrous cycle. A transient increase in testosterone concentrations was only observed after ovulation, on Day 18 (Fig. 7), when the rats were in metoestrus, as reported by others.^50^ In contrast, above a certain threshold of both AMH and androgen levels, which is difficult to determine due to their variability in both Wistar and GK rats,^19^ ovulation could not occur. This may explain why GK rats did not ovulate in the second treatment despite the reduction in bioavailable AMH and androgen levels (Fig. 4), nor after ovulation from Day 18 to Day 31 in the third protocol. Additional studies are required to determine the respective mechanisms of action of androgens and AMH in blocking ovulation in GK rats at the central and ovarian levels.

The role of hyperandrogenism in the ovulation defects observed in PCOS-like mouse models was elegantly demonstrated by comparing global and cell-specific androgen receptor (AR) knockout mouse lines treated with dihydrotestosterone (DHT) or with AMH. These studies showed that deletion of AR in granulosa or theca cells did not prevent the development of a PCOS-like phenotype in prepubertal androgenised mice, in contrast to a knock-out (KO) of AR in neurons,^51^ demonstrating the central action of androgens. In addition, Ho et al.^52^ reported that the AMH-induced PCOS-like reproductive deficits in prenatally AMH-treated female mice were abolished in kisspeptin-specific ARKO mice, demonstrating that these effects of AMH are mediated by androgens acting on kisspeptin neurons during the prenatal period. However, consistent with an important role for AMH in addition to androgens in the ovulation process, serum AMH levels are correlated with PCOS severity in terms of ovulation^12^ and in women with PCOS, AMH levels are lower in patients with a hyperandrogenic normo-ovulatory phenotype than in women with a non-hyperandrogenic oligo-anovulatory phenotype.^53^ AMH may therefore act on ovulation through androgens, but also directly on both the LH surge at the level of the hypothalamic–pituitary complex, and terminal folliculogenesis at the level of ovary. Further studies will investigate the effects of rMab22A2 on the other PCOS-like abnormalities observed in GK rats, in particular the metabolic and central alterations.

The following limitations of this study should be noted. First, even if the Mab22A2 antibody recognises human AMH and prevents its signalling in vitro, its ability to block the biological effects of AMH in human cells has not been demonstrated. Similarly, we have not controlled whether the rMab22A2 still binds to AMH and specifically to rat AMH. However, the decrease in AMH levels after rMab22A2 injection in GK rats is likely due to the fact that rMab22A2 sequesters rat endogenous AMH, preventing the recognition of rat AMH by one of the two anti-AMH antibodies of the ELISA. The ELISA results will then show reduced detectable AMH levels and probably reduced bioavailable AMH levels, but this cannot be represented as rMab22A2 normalising AMH levels. The third protocol clearly has room for improvement. At this stage, since we cannot distinguish between the duration of treatment or the antibody doses, which parameter is more important, an important issue will be to evaluate the efficiency of each rMab22A2 injection and the stability of rMab22A2 in relation to serum AMH. Although the protocols tested did not induce serious side effects during the period studied, it would also be necessary to monitor the safety of the treatment. Our results should be interpreted in the context of these limitations, which may affect the generalisability of our findings to the more complex management of women with PCOS.

Despite these limitations, we describe in this article an antibody against AMH, Mab22A2, which is able to restore normal bioavailable AMH concentrations and androgen levels, as well as an ovulation in the GK model of PCOS. Fundamentally, our data confirm the role of AMH in terminal folliculogenesis and they suggest that a fine-tuned balance between AMH and androgen levels is required for an ovulation to occur. Most importantly, our results provide proof of concept that an immunotherapy against AMH could reverse the major reproductive dysfuntions observed in PCOS, opening up promising avenues for the treatment of women with PCOS.

## Contributors

C.R. and N.d.C. conceived and designed the study, performed most of the experiments, analysed and verified the data, prepared the figures, wrote the manuscript and were responsible for the decision to submit the manuscript. R.C. participated in the design, experiments and analyses of the in vitro studies, and helped to discuss the paper. F.F. analysed liquid chromatography-mass spectrometry experiments performed by T.P. S.T., S.G. and D.B. performed several in vivo experiments. A.B. checked all the statistical analyses. J.M. and B.F. critically revised the final manuscript. All the authors read and approved the final version of the manuscript.

## Data sharing statement

The data supporting the findings of this study and the protocols are available upon reasonable request to the corresponding author.

## Declaration of interests

Nathalie di Clemente and Richard Cate are inventors in: Anti-Müllerian hormone (AMH) neutralizing Antibodies And Uses Thereof. EP 3353205 A1, US 10,774,142. All other authors confirm that they have no competing interests.

## Appendix A. Supplementary data

Supplementary Figs. S1 and S2 and Tables S1 and S2 **Supplementary Fig. S1: KK1 cells respond to AMH after transfection with AMHR2 cDNA, and Mab22A2 neutralises AMH effect on Smad1 signalling.** (**a**) KK1 granulosa cells were co-transfected with Smad5 or Smad1 reporter system, mouse or human AMHR2 cDNA and pRLTK plasmid. Luciferase activity was assessed after 24 h of treatment with either control medium or C-term AMH (2 nM). (**b**) KK1 granulosa cells were co-transfected with Smad1 reporter system, mouse AMHR2 cDNA and pRLTK plasmid. Luciferase activity was assessed after 24 h of treatment with either control medium or AMH procomplex (7.14 nM), pre-incubated or not 1 h with an excess of Mab22A2 (350 nM) or Mab10.6 (700 nM). Results were expressed as the ratio of Firefly to Renilla luciferase activity. Each point represents a replicate from at least 3 experiments. Data are presented as a scatter plot bar with mean with 95% CI. P-values are from the Kruskal-Wallis test followed by the Dunn’s multiple comparison test. **Supplementary Fig. S2: Chronic low-dose rMab22A2 treatment has no effect on adipose tissue repartition and on leptin levels in GK rats.** Diagram showing the protocol of eight chronic injections of 100 μg of rMab22A2, IgG2 or PBS. The anovulatory status of 5-7 month old GK rats was checked by daily analysis of their vaginal smears for 15 days before they were treated with 8 intraperitoneal (IP) injections of 100 μg of rMab22A2 every 2 days. (**b**) Amounts of fat mass and lean mass determined by EcoMRI™ 100 two days before (white bars) and 10 days after the first injection (Day 10, orange bars), and expressed as a percentage of the body weight of rMab22A2- or PBS-treated GK rats. (**c**) Blood samples were taken from the tail two days before (white bars) and 10 days after the first injection (Day 10, orange bars). Data are represented as a scatter plot bar, with mean 95% CI. P-values are from paired t-test (b) or sign test (PBS group in c). *n* represents the number of animals in each group.

Western Blotting Smad1 5 8

Western Blotting actin
