# Supplementary material for: A blocking antibody against anti-Müllerian hormone restores ovulation and normal androgen levels in a spontaneous rat model of polycystic ovary syndrome
Source: eBioMedicine. 2025 Apr 18;115:105716. doi: 10.1016/j.ebiom.2025.105716 (PMC12032919; doi:10.1016/j.ebiom.2025.105716)
Supplement: Supplementary Figs. S1 and S2 and Tables S1 and S2 — Supplementary Fig. S1: KK1 cells respond to AMH after transfection with AMHR2 cDNA, and Mab22A2 neutralises AMH effect on Smad1 signalling. (a) KK1 granulosa cells were co-transfected with Smad5 or Smad1 reporter system, mouse or human AMHR2 cDNA and pRLTK plasmid. Luciferase activity was assessed after 24 h of treatment with either control medium or C-term AMH (2 nM). (b) KK1 granulosa cells were co-transfected with Smad1 reporter system, mouse AMHR2 cDNA and pRLTK plasmid. Luciferase activity was assessed after 24 h of treatment with either control medium or AMH procomplex (7.14 nM), pre-incubated or not 1 h with an excess of Mab22A2 (350 nM) or Mab10.6 (700 nM). Results were expressed as the ratio of Firefly to Renilla luciferase activity. Each point represents a replicate from at least 3 experiments. Data are presented as a scatter plot bar with mean with 95% CI. P-values are from the Kruskal-Wallis test followed by the Dunn’s multiple comparison test. Supplementary Fig. S2: Chronic low-dose rMab22A2 treatment has no effect on adipose tissue repartition and on leptin levels in GK rats. Diagram showing the protocol of eight chronic injections of 100 μg of rMab22A2, IgG2 or PBS. The anovulatory status of 5-7 month old GK rats was checked by daily analysis of their vaginal smears for 15 days before they were treated with 8 intraperitoneal (IP) injections of 100 μg of rMab22A2 every 2 days. (b) Amounts of fat mass and lean mass determined by EcoMRI™ 100 two days before (white bars) and 10 days after the first injection (Day 10, orange bars), and expressed as a percentage of the body weight of rMab22A2- or PBS-treated GK rats. (c) Blood samples were taken from the tail two days before (white bars) and 10 days after the first injection (Day 10, orange bars). Data are represented as a scatter plot bar, with mean 95% CI. P-values are from paired t-test (b) or sign test (PBS group in c). n represents the number of animals in each group. [file mmc1.pdf]

List of captions for each Supplementary file

Supplementary Fig. S1: KK1 cells respond to AMH after transfection with AMHR2 cDNA, and Mab22A2 neutralises AMH effect on Smad1 signalling.

Supplementary Fig. S2: Chronic low-dose rMab22A2 treatment has no effect on adipose tissue repartition and on leptin levels in GK rats.

Supplementary Table S1: Sequence of rat primers used for RT-qPCR.

Supplementary Table S2: Mating table for 5 months old Wistar rats.

Supplementary Figure S1

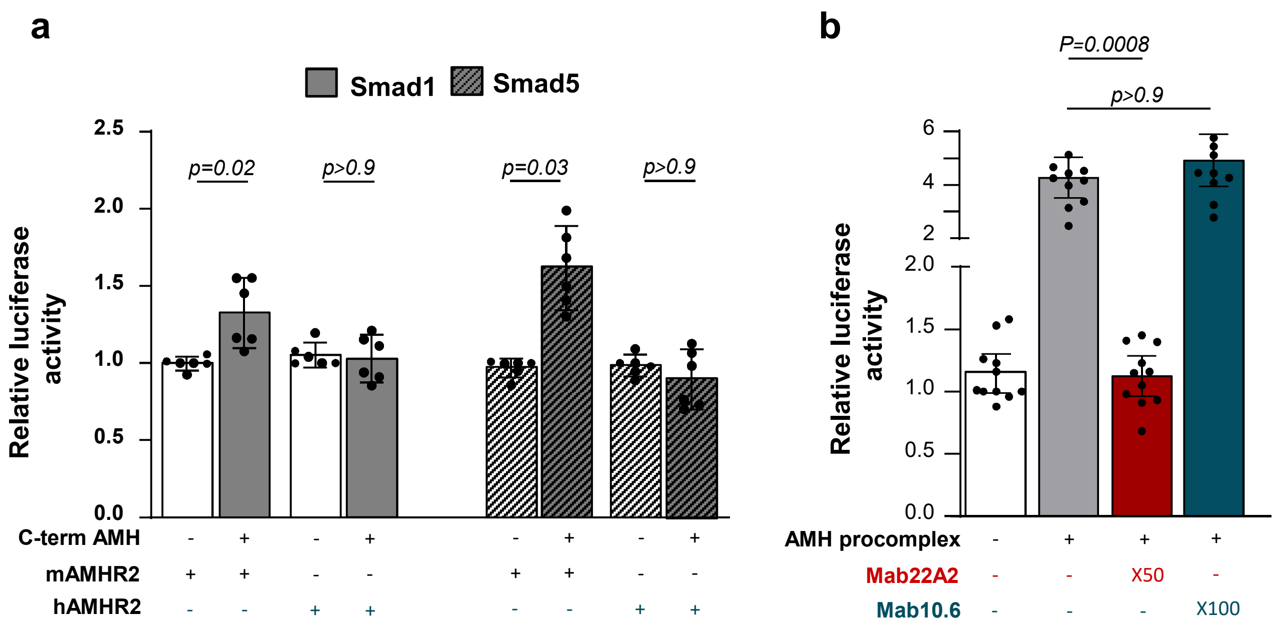

Supplementary Figure S2

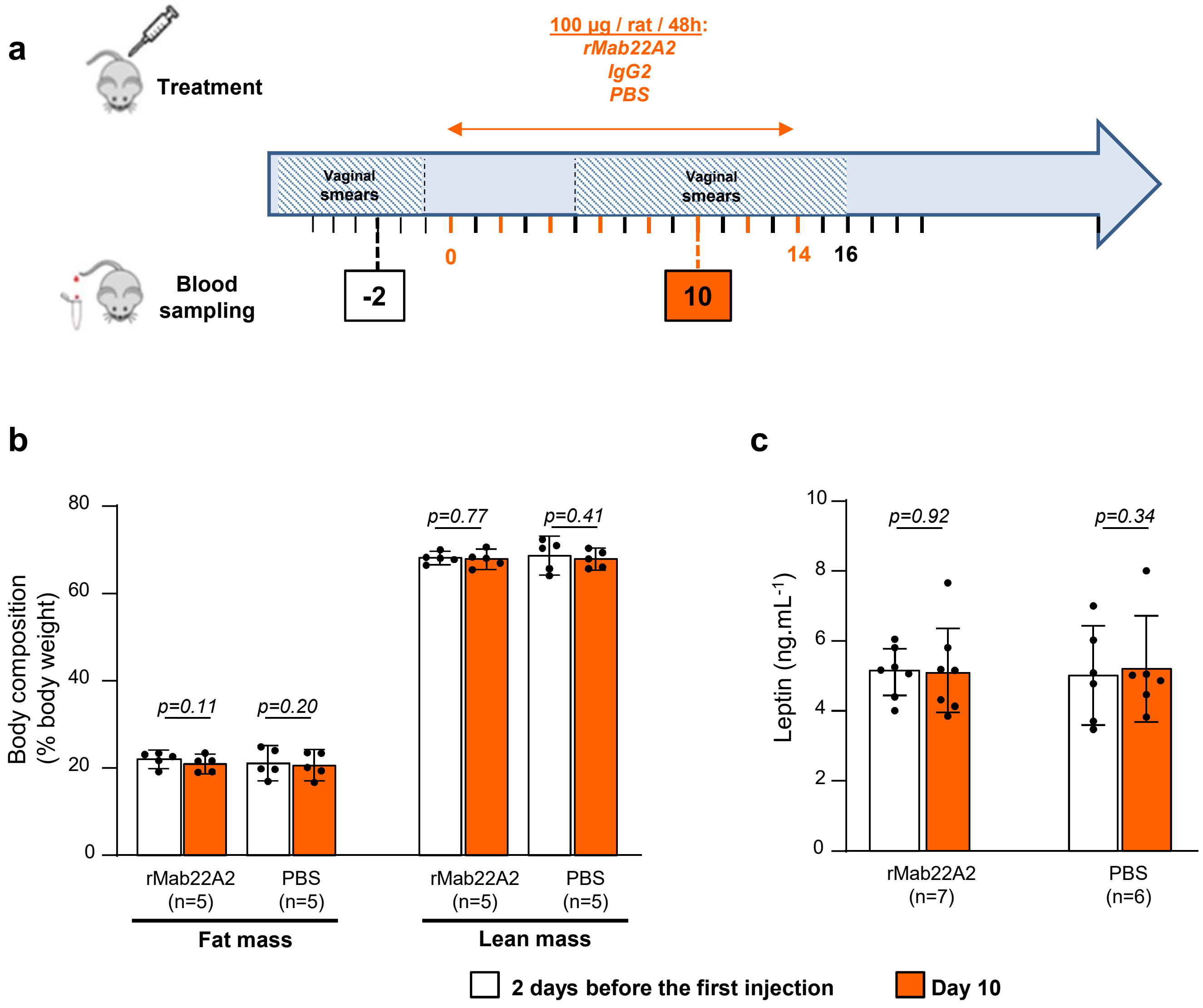

Supplementary Table S1. Sequence of the primers used for real time RT-PCR experiments

Gene	Nucleotide sequence 5' --> 3'		reference sequence	Length (bp)
	Forward	Reverse		
Id1	ACCCTCTCCACCTTTCAG	AAATCTGAGAAGCACGAA	NM_012797	121
Hprt	GGTCCATTCCTATGACTGTAGATTTT	CAATCAAGACGTTCTTTCCAGTT	NM_012583	126
Smad6	TATTCTCGGCTGTCTCCT	CCTCGGTTTCAGTGTAAGA	NM_001109002	78
Smad7	AAGATTGAAGCAGCCTAA	CGTATCCACGAGTTACAT	NM_030858	131
StAR	AGGCTGGAAGAAGGAAAGCC	TCTGTCCATGGGCTGGTCTA	NM_031558	114
Cyp19a1	GGAAATCCACACTGTTGTTGG	TGAAGTTTTCCACCACTTTCAA	NM_017085	76

Supplementary Table S2: Mating table for 5 months old Wistar rats

Number of rats	Age of birth	Age at mating	Number of mating days	Number of pregnant rats
8	11/18/2023	04/24/2024	1	3
2	06/16/2023	11/15/2024	4	2
1	06/17/2023	11/15/2024	4	1
6	06/18/2023	11/15/2024	4	3
1	06/19/2023	11/15/2024	4	0
