# Supplementary figures and images for: A blocking antibody against anti-Müllerian hormone restores ovulation and normal androgen levels in a spontaneous rat model of polycystic ovary syndrome
Source: eBioMedicine. 2025 Apr 18;115:105716. doi: 10.1016/j.ebiom.2025.105716 (PMC12032919; doi:10.1016/j.ebiom.2025.105716)

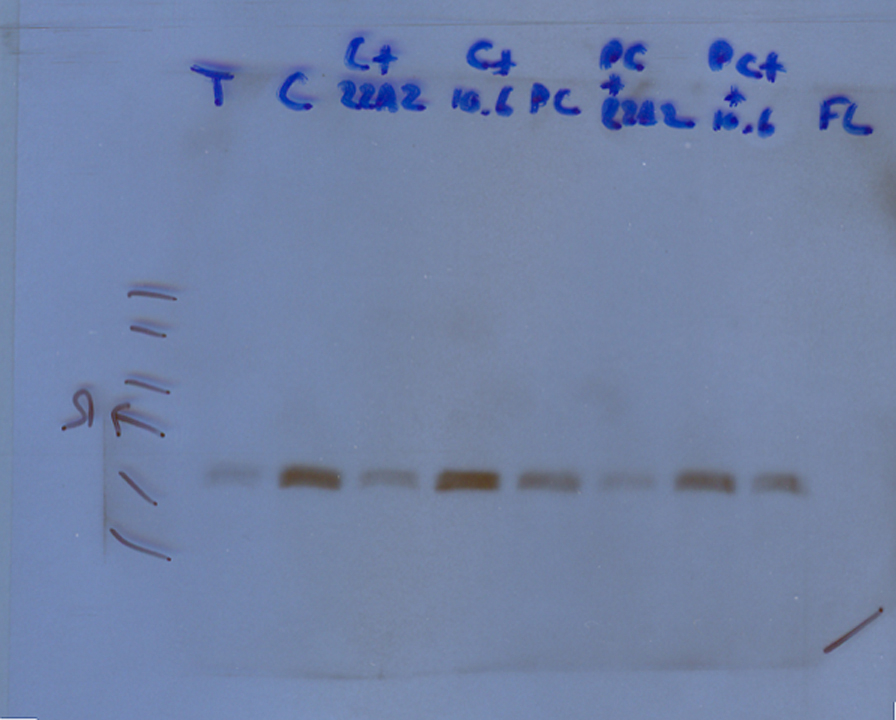

Supplement: Western Blotting Smad1 5 8 [file figs1.jpg]

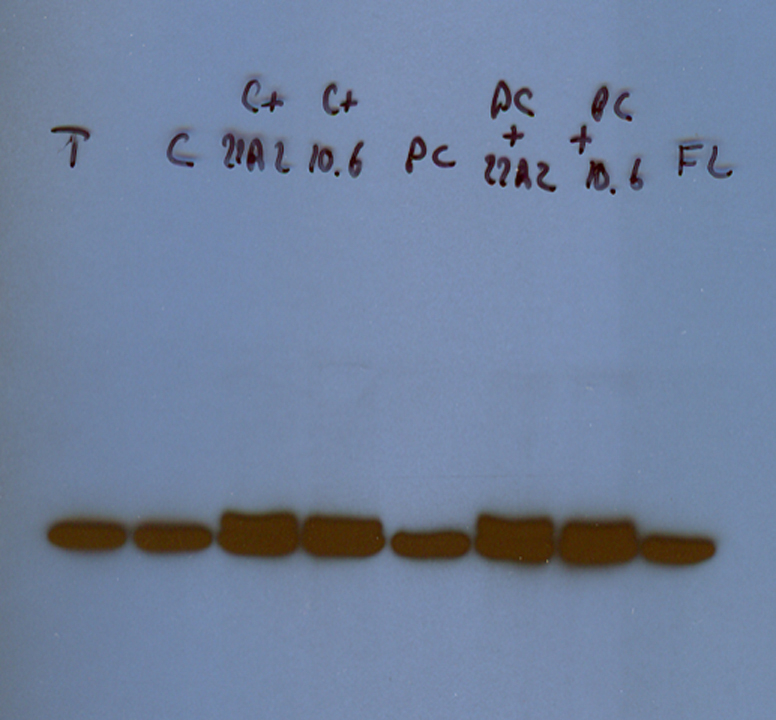

Supplement: Western Blotting actin [file figs2.jpg]
